# Utility of CSF biomarkers in psychiatric disorders: a national multicentre prospective study

**DOI:** 10.1186/s13195-016-0192-z

**Published:** 2016-06-13

**Authors:** Claire Paquet, Eloi Magnin, David Wallon, Anne-Cécile Troussière, Julien Dumurgier, Alain Jager, Frank Bellivier, Elodie Bouaziz-Amar, Frédéric Blanc, Emilie Beaufils, Carole Miguet-Alfonsi, Muriel Quillard, Susanna Schraen, Florence Pasquier, Didier Hannequin, Philippe Robert, Jacques Hugon, François Mouton-Liger, For ePLM network and collaborators

**Affiliations:** INSERM UMR-S942 Université Paris Diderot, 75010 Paris, France; Centre Mémoire (CMRR) Paris Nord Ile de France, Groupe Hospitalier Lariboisiere FW Saint-Louis, APHP, Université Paris Diderot, 200, rue du Faubourg Saint Denis, 75010 Paris, France; Unité d’Histologie et de Biologie du Vieillissement, Groupe Hospitalier Lariboisiere FW Saint Louis APHP, Université Paris Diderot, 75010 Paris, France; Centre Mémoire (CMRR) de Besançon, Hôpital Universitaire de Besançon, 25000 Besançon, France; Centre Mémoire (CMRR) and INSERM UMR1079, Hôpital Universitaire de Rouen, 76000 Rouen, France; Centre Mémoire (CMRR) de Lille, Université Lille Nord de France, UDSL, 59000 Lille, France; Centre de Neurologie, 6 place Luxembourg, 57100 Thionville, France; INSERM UMR-S1144 and Service de Psychiatrie, Groupe Hospitalier Lariboisiere FW Saint-Louis, APHP, Université Paris Diderot, 75010 Paris, France; Service de Biochimie, Groupe Hospitalier Lariboisiere FW Saint-Louis, APHP, Université Paris Diderot, 75010 Paris, France; Centre Mémoire (CMRR) de Tours, Hôpital Universitaire Tours, 37000 Tours, France; Université de Strasbourg, CNRS, Laboratoire ICube UMR 7357 and Fédération de Médecine Translationnelle de Strasbourg (FMTS), 67000 Strasbourg, France; Centre Mémoire (CMRR), Hôpital Universitaire de Strasbourg, Département de Gériatrie, Hôpital de Jour Gériatrique, 67000 Strasbourg, France; Service de Biochimie, Hôpital Universitaire de Besançon, 25000 Besançon, France; Service de Biochimie, Hôpital Universitaire de Rouen, 76000 Rouen, France; Université Lille, CHU-Lille, Inserm, UF de Neurobiologie, CBPG, Lille, France; CMRR COBTEK research unit, Université de Nice Sophia Antipolis, 06100 Nice, France

**Keywords:** Cerebrospinal fluid, Psychiatric disease, Biomarkers, Alzheimer’s disease, Clinical practice

## Abstract

**Background:**

Affective and psychotic disorders are mental or behavioural patterns resulting in an inability to cope with life’s ordinary demands and routines. These conditions can be a prodromal event of Alzheimer’s disease (AD). The prevalence of underlying AD lesions in psychiatric diseases is unknown, and it would be helpful to determine them in patients. AD cerebrospinal fluid (CSF) biomarkers (amyloid β, tau and phosphorylated tau) have high diagnostic accuracy, both for AD with dementia and to predict incipient AD (mild cognitive impairment due to AD), and they are sometimes used to discriminate psychiatric diseases from AD. Our objective in the present study was to evaluate the clinical utility of CSF biomarkers in a group of patients with psychiatric disease as the main diagnosis.

**Methods:**

In a multicentre prospective study, clinicians filled out an anonymous questionnaire about all of their patients who had undergone CSF biomarker evaluation. Before and after CSF biomarker results were obtained, clinicians provided a diagnosis with their level of confidence and information about the treatment. We included patients with a psychiatric disorder as the initial diagnosis. In a second part of the study conducted retrospectively in a followed subgroup, clinicians detailed the psychiatric history and we classified patients into three categories: (1) psychiatric symptoms associated with AD, (2) dual diagnosis and (3) cognitive decline not linked to a neurodegenerative disorder.

**Results:**

Of 957 patients, 69 had an initial diagnosis of a psychiatric disorder. Among these 69 patients, 14 (20.2 %) had a CSF AD profile, 5 (7.2 %) presented with an intermediate CSF profile and 50 (72.4 %) had a non-AD CSF profile. Ultimately, 13 (18.8 %) patients were diagnosed with AD. We show that in the AD group psychiatric symptoms occurred later and the delay between the first psychiatric symptoms and the cognitive decline was shorter.

**Conclusions:**

This study revealed that about 20 % of patients with a primary psychiatric disorder diagnosis before undergoing a CSF exploration for cognitive disorder displayed a CSF biomarker AD profile. In memory clinics, it seems important to consider AD as a possible diagnosis before finalizing a diagnosis of a psychiatric disorder.

## Background

An association between Alzheimer’s disease (AD) and thymic or psychotic disorders has been reported, suggesting that they could be considered either as a risk factor for [1, 2] or as a prodromal condition of AD [3], or sometimes in a differential diagnosis [4, 5]. Until recently, the diagnosis of AD was based on the McKhann’s criteria [6] in clinical practice, in recent cohorts [7] and in international clinical trials [8, 9]. However, in neuropathological studies [10, 11], positron emission tomography (PET) amyloid imaging [9, 12] and cerebrospinal fluid (CSF) biomarker studies, researchers have found that atypical AD forms are more frequently observed [13, 14], and the diagnosis appears to be a challenge when it is based only on presenting clinical features without specific biomarkers [15, 16]. Taking into account these findings, new criteria were proposed for AD [15], mild cognitive impairment (MCI) [3] and pre-clinical stage [17], including imaging and/or biological markers. AD CSF biomarkers are the only markers that reflect the AD neuropathological lesions associating tau and amyloid β [11, 18–20]. Positive CSF biomarkers imply that patients have AD brain lesions with or without clinically detectable AD features. These CSF biomarkers are widely used and validated in AD in clinical research [21, 22] and in clinical practice [23]. Importantly, these markers are normal in several other disorders, such as depression. However, no study to date has explored the usefulness of CSF biomarkers in patients diagnosed with psychiatric disorders and cognitive symptoms in daily clinical practice. In this large, multicentre, prospective observational cohort study, our aims were (1) to determine, in this subgroup with an initial diagnosis of a psychiatric disorder, the proportion of patients diagnosed with psychiatric disorders who had CSF AD profiles; and (2) to analyse the impact of CSF biomarker results on the final diagnosis in this subgroup.

## Methods

### Study subjects

This study was designed to investigate the impact of CSF biomarker results on the diagnoses made by experienced clinicians in their routine daily practice. The clinicians’ participation was voluntary. All senior clinicians who wanted to participate replied to an email invitation. When a clinician considered a patient eligible for CSF biomarkers, he filled out a questionnaire about the patient, for whom collected information was anonymous. This questionnaire was created by a team that included neurologists and biologists, and then it was evaluated in clinical practice during a 1-month period by clinicians.

All recruiting centres are either secondary or tertiary memory centres. These centres have used the same clinical and biochemical procedures and internationally validated criteria for the diagnosis of AD [6] and all other forms of dementia. Patients underwent a thorough examination, including clinical, neurological and neuropsychological evaluations and brain imaging. As recommended by the Haute Autorité de Santé (French Health Authority), CSF biomarkers can be used in clinical practice in cases of atypical clinical presentation and/or rapidly progressive cognitive decline, and/or in cases of diagnostic uncertainty, particularly in young patients. Consequently, in France, CSF AD biomarkers are used in clinical practice mainly in tertiary memory centres to which patients with depression or psychiatric disorders are also referred to determine a possible AD diagnosis.

The questionnaire was divided into two parts:

1. The first part was completed by ticking boxes before the lumbar puncture (LP) was performed. Clinicians were asked to mention the other explorations performed before LP, and they were required to indicate their most suspected diagnosis (initial diagnosis). Clinicians were asked to rate the level of confidence of their initial diagnosis on a numerical visual scale ranging from 0 to 10. Diagnosis hypotheses were established by the clinician in charge of the patient.
2. The second part was filled out after LP and CSF biomarker results were obtained. Clinicians were required to indicate AD CSF biomarkers results as “biological AD profile” or “not indicative of AD biological profile” or “non-contributory” (including CSF with technical problems, intermediate CSF or CSF without interpretation). Clinicians were asked to write their final diagnosis according to a list of diseases and to rate their level of confidence with a similar numerical visual scale and to indicate possible changes concerning the medications [16].

CSF biomarker tests were performed in local laboratories for all subjects. All biochemical teams were involved in two national cohorts and shared the same standards for CSF analysis and interpretation [24–28]. In addition, the laboratories performed two external quality controls: one run by a working group of the Société Française de Biologie Clinique (French Society of Clinical Biology) and the other by the Alzheimer’s Association [29]. Our aim was to get the most reliable and realistic opinions of clinicians in routine clinical practice without interfering with their diagnosis process or with their conclusions regarding the interpretation of CSF AD biomarkers.

For the present study, we explored the subgroup with an initial diagnosis of a psychiatric disorder before CSF biomarker results were obtained. This subgroup was designated as the study population. The final diagnosis corresponded to the clinician’s final conclusion indicated on the questionnaire at the end of all explorations. We considered that there was a “changed diagnosis” each time the initial and final diagnoses were different. As recommended in France, all diagnoses were made by a multidisciplinary team of the research memory centres. When possible, the psychiatric history and the precise psychiatric diagnosis were retrospectively collected at the end of the study from local psychiatrists and classified according to the *Diagnostic and Statistical Manual of Mental Disorders, Fourth Edition, Text Revision*.

Retrospectively, to complete analyses, clinicians were asked to answer the following questions for each patient: (1) regarding the onset of the psychiatric symptoms, new onset of psychiatric symptoms or a long history of psychiatric symptoms; (2) age at the onset of psychiatric symptoms; (3) information about the cognitive profile (amnestic, dysexecutive syndrome, language disturbance, apraxia, global dysfunction); and (4) the evolution of the patient’s cognitive profile (improvement, stable, declining). Finally, according to clinical information, we classified each patient into one of three categories: (1) psychiatric symptoms associated with AD, (2) cognitive impairment not linked to neurodegenerative disorder or (3) dual diagnosis (patient with a primary psychiatric disease who then subsequently developed neurodegenerative disease).

To evaluate the external validity of our work, we compared our study population with the population registered in the Banque Nationale Alzheimer (BNA) during the same period "(DGOS : Direction Générale de l'Offre de Soins) Centre Hospitaleir Universitaire de Nice". The BNA records each consultation performed in all memory clinics in France [30]. We considered that there was a changed diagnosis in the BNA each time the initial diagnosis was modified during follow-up.

### Statistical analysis

Baseline characteristics of our study population were presented and compared with our overall population and with the BNA population (including overall population and the psychiatric disorders subgroup). We compared our overall and study populations with the BNA population to determine if our populations were representative of the national population of patients.

Concerning CSF biomarkers results, as CSF AD biomarkers reflect neuropathological AD lesions, we classified patients in the biological AD category whenever the AD CSF box was checked and in the non-AD category when the AD CSF box was not checked. For the analysis concerning the final diagnosis, patients were classified as AD or non-AD. These groups were compared using χ^2^ statistics for categorical variables and the *t* test (parametric or non-parametric) for age.

Further analyses were performed with retrospective complementary data about the history of the psychiatric disorder, the age of onset and the delay between the first psychiatric symptom and cognitive decline. Because of the lack of statistical power, the cognitive profile and the evolution were described but not compared. Analyses were performed using SAS 9.2 software (SAS Institute, Cary, NC, USA).

## Results

**Fig. 1:**
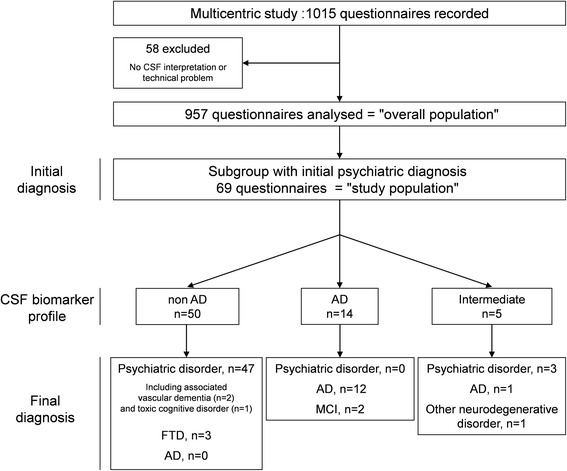
Flowchart of the operational mode for patient inclusion. *AD* Alzheimer’s disease, *CSF* cerebrospinal fluid, *FTD* frontotemporal dementia, *MCI* mild cognitive impairment

For the last two years of the present study we recorded the results of 1015 questionnaires about patients who underwent LP for cognitive disorders at 29 memory clinics (including 61 senior neurologists, 65 senior geriatricians and 2 senior psychiatrists). Fifty-eight questionnaires (5.7 %) were excluded for missing data. A total of 957 questionnaires were ultimately analysed and were defined as the overall population. In this overall population, 69 (7.3 %) patients were diagnosed with psychiatric disorders as their main initial diagnosis (anxiety and/or depression 62.3 %, bipolar disorder 17.4 %, psychosis 14.5 %, others 5.8 %). This subgroup was defined as our study population. A flowchart of the study population is presented in Fig. 1. Characteristics of the overall population compared with BNA (overall and patients with psychiatric disorders) are shown in Table 1. The proportion of psychiatric disorders was comparable in our overall and BNA populations (*p* = 0.29). Populations were different in terms of age but not sex distribution; there was a female predominance. Comparably to our overall population, most of the patients of the study population (*n* = 65, 92.9 %) were evaluated with other examinations before undergoing LP (*p* = 0.27).

**Table 1:** Characteristics of the study population before cerebrospinal fluid biomarker diagnosis

	Study population			BNA population			BNA/study population comparison
	Overall	Psychiatric disorder	*p* Value	Overall	Psychiatric disorder	*p* value	*p* Value
Number of patients	1015	69 (6.79)		317,515	24,561 (7.74)		0.29
Age, years, mean (SD)	69.2 (10.03)	64.57 (11.57)	0.0018	76.86 (12.18)	71.43 (13.82)	<0.0001	<0.0001
Female sex, *n* (%)	504 (49.7)	43 (61.4)	0.056	199,085 (62.7)	17,661 (71.91)	<0.0001	0.46
Final diagnosis of AD, *n* (%)	395 (38.9)	13 (18.8)	0.009	70,651 (22.25)	536 (2.18)	<0.0001	<0.0001
Changed diagnosis, *n* (%)	231 (24.1)	19 (27.5)	0.62		1821 (7.41)		<0.0001
Improvement in diagnosis confidence level, %	19.6	25.2	0.027				
Changed treatment, *n* (%)	437 (43.1)	24 (34.8)	0.13				

*AD* Alzheimer’s disease, *BNA* Banque Nationale Alzheimer

In comparison with our overall population, the study population was younger (*p* = 0.0018) and significantly less often diagnosed with AD (*p* = 0.009), but the rates of changed diagnosis were comparable (Table 1). In comparison with the psychiatric BNA population (including patients with and without CSF biomarkers), our study population was significantly younger (*p* < 0.0001), more frequently diagnosed with AD (*p* < 0.0001) and had a higher rate of changed diagnosis (*p* < 0.0001).

Figure 1 details the repartition of CSF biomarker results and final diagnoses. According to the final diagnoses, patients of the study population were classified as having AD (*n* = 13, 18.8 %), MCI (*n* = 2, 2.9 %), psychiatric disorder (*n* = 50, 79.7 %), frontotemporal dementia (FTD) (*n* = 3, 4.3 %) and other neurodegenerative disease (*n* = 1, 1.4 %). Subjects with a final diagnosis of AD were not different in terms of age and sex from non-AD patients. In most cases (*n* = 50, 72.4 %), CSF results were concordant with the initial diagnosis, with no changed diagnosis. Changed diagnosis after CSF biomarker results occurred when CSF biomarker results were discordant with the initial diagnosis. Clinicians changed their diagnoses for all patients who had CSF AD profiles (*n* = 12 for AD, *n* = 2 for MCI) and for two patients with intermediate biological profiles. Among the five patients with intermediate profiles, we found the following diagnoses: AD (*n* = 1), other degenerative disorder without precision (*n* = 1) and initial psychiatric diagnosis (*n* = 3). There were 3 changed diagnoses among the 50 patients with non-AD CSF profiles; these 3 patients were diagnosed with FTD. Finally, clinicians concluded on a non-AD diagnosis in 100 % of cases when CSF biomarker results were not in favour of AD.

According to the numerical scale of confidence, the mean value for clinician certainty of the final diagnosis was significantly increased by the use of CSF biomarkers: 8.07 ± 1.37 after CSF results versus 6.04 ± 1.41 before (*p* < 0.0001). These results were comparable to those for our overall population. The mean confidence was similar for AD and non-AD patients (*p* = 0.35). Concerning the treatment, in the study population, 24 (30.4 %) patients had a modification of the treatment, including prescription or stopping acetylcholinesterase inhibitors (*n* = 7 and *n* = 5, respectively) or other modifications (*n* = 12).

**Fig. 2:**
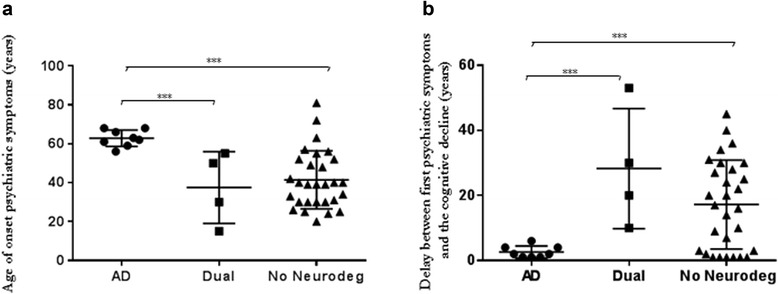
Comparison of the three categories of patients in the study: (1) psychiatric symptoms associated with Alzheimer’s disease (AD), (2) cognitive impairment not linked to neurodegenerative disease (No Neurodeg) and (3) dual diagnosis (patient with a primary psychiatric disease who subsequently developed AD) (dual). **a** Differences in age of onset of psychiatric symptoms between the three categories. **b** Differences in delay between the first psychiatric symptoms and cognitive decline in the three categories ***: *p*<0.001

As explained in the Methods section above, we could determine the precise chronological link between cognitive decline, psychiatric symptoms and evolution of the disease in 41 patients. According to the clinical information we gathered, we classified patients into three categories: (1) 8 patients with psychiatric symptoms associated with AD, among whom 7 had a CSF biomarker AD profile and 1 had an intermediate profile; (2) 29 with cognitive impairment not linked to a neurodegenerative disorder, including 27 with non-AD CSF biomarker profiles and 2 with intermediate profiles; and (3) 4 with a dual diagnosis (association of a psychiatric disease and a neurodegenerative disorder), including 3 with CSF AD profiles and 1 with a non-AD profile. The analysis showed that the first psychiatric symptoms occurred significantly later in life in the AD group than in the group with cognitive impairment not linked to a neurodegenerative disorder (*p* = 0.0002) and the group with a dual diagnosis (*p* = 0.004). The delay between the first psychiatric symptoms and the onset of the cognitive decline was shorter in the AD group than in the group with cognitive impairment not linked to a neurodegenerative disorder (*p* = 0.0064) and the group categorized with a dual diagnosis (*p* = 0.002). No difference was observed between the last two categories. The results of all of these comparisons are shown in Fig. 2.

Most of the patients in the AD group (seven of eight) and in the dual diagnosis group (three of four) had a cognitive decline predominantly due to amnestic impairment. We found 12 patients with amnestic decline in the group of patients with cognitive decline not linked to neurodegenerative disorder. The predominant cognitive profile in this last group was dysexecutive syndrome (*n* = 17). After 3 years of follow-up, seven patients in the AD group were in cognitive decline and one was still stable. In the dual diagnosis group, one patient was stable and three were in decline. In the group of patients with cognitive decline not linked to neurodegenerative disorder, the evolution was variable: 15 were stable, 2 were fluctuant, 2 were improved and 5 had gotten worse.

## Discussion

In this large multicentre prospective study reflecting daily practice, we determined the biological AD profiles of patients with a primary psychiatric diagnosis. We present the potential impact of this diagnosis on the therapeutic management of this population. Moreover, we highlight the differences in the age of onset and the delay between the first psychiatric symptom and the age of cognitive decline between patients with AD, those with a dual diagnosis and those with cognitive decline not linked to a neurodegenerative disorder.

The design of this prospective study was based on consecutive inclusion and local clinical diagnosis and biochemical examination interpretation, which made it possible for us to make a realistic estimation of the links between CSF biomarker results and the clinical diagnosis of patients with psychiatric symptoms. In our overall population, comparably to our BNA population, the percentage of psychiatric diagnosis was low (7.3 %), but the proportion of patients with probable AD in this group was notable (about 20 %). The impact of CSF biomarker results was clear with regard to the magnitude of changed diagnoses, with treatment modification in more than 30 % of cases. In addition, a significant improvement in clinicians’ confidence in their diagnoses was noted. These results illustrate the benefit of CSF biomarker assessment for patients with psychiatric and cognitive symptoms.

Concerning age, we noted an age difference between our overall population, whereas there was no difference between AD and non-AD within the study group. These observations could be linked with the fact that early AD can be associated with psychiatric symptoms. These results emphasize that, in memory centres, the diagnosis cannot be based only on indications given by the patient’s age and that often a precise history of the psychiatric symptoms and further explorations is needed. Nevertheless, our study does not provide information establishing a direct pathophysiological link between AD and psychiatric symptoms; thus, we could not confirm that psychiatric symptoms reveal AD pathology. We also found age differences between our populations and the BNA populations, possibly due to the fact that BNA records all patients from all French memory clinics [31], while our study was carried out on a voluntary basis, and consequently we included a limited number of centres that perform CSF biomarker evaluations. Our overall population and our study populations were significantly younger and more frequently diagnosed with AD than the BNA overall and psychiatric populations [16]. This difference is in accordance with the recommendations by French authorities and with the current use of CSF biomarkers that are used mainly in younger patients or in patients with atypical presentations [23]. Furthermore, CSF AD biomarkers are more discriminant in younger than in older patients with AD reinforcing the tendency to assess younger individuals [32]. Our population had a significantly higher rate of patients with AD than the BNA population, probably because CSF biomarker evaluations are performed when a neurodegenerative disease is strongly suspected. In the BNA population, all patients are recorded, including patients without any biomarkers.

Our CSF biomarker results are in accord with those in the study by Blennow et al., who showed that in patients with pure psychiatric disorders, the CSF biological profile is in favour of neither AD nor an intermediate profile, which makes the results very informative in differentiating AD from non-AD patients [33]. Surprisingly, although pure depression revealing dementia with Lewy bodies (DLB) has been described previously [34], we did not find any patients with DLB. This result could be explained by the small cohort, the translational methodology with a short follow-up in the retrospective part of the study, the lack of neuropathological confirmation, and possibly misdiagnoses. We do know that DLB is underdiagnosed, since only 32 % of the patients are diagnosed [35] and DLB is frequently confused with AD or psychiatric diseases. However, patients with DLB display some specific behavioural disorders, such as feeling of presence, illusions or visual hallucinations [36], that were not described in our population.

In the complementary analysis, we made some very interesting observations. In the AD group, the first psychiatric symptoms occurred later in life than in the non-AD group, confirming that new onset of psychiatric symptoms later in life may be more suggestive of neurodegeneration. In the AD group, the delay between the first psychiatric symptoms and cognitive decline was relatively short (<5 years), while it is very heterogeneous in the two other categories. We could hypothesise that, in the AD group, the psychiatric symptoms could be among the first symptoms of AD linked to the regional AD lesions. In contrast, in the other groups, the cognitive decline was associated either with neurodegeneration or with pure psychiatric disease or a chronic neuronal dysfunction of unknown pathophysiology. Figure 2 illustrates these results. Note that all the results in the AD category are very homogeneous: The first psychiatric symptoms always occur late in life, and the delay between the first psychiatric symptoms and cognitive decline is always short. The relationship seems more heterogeneous in the two other categories. These observations could suggest that there is a close link between the two. However, further, larger studies are needed to confirm these observations.

In our results, clinical diagnosis and CSF biomarker results were concordant in about two-thirds of cases, a finding close to those of neuropathological studies [10, 11]. Unlike post-mortem histological studies that reclassify the diagnosis after several years of evolution, a reclassification based on CSF biomarkers occurred quite often at the beginning of follow-up and may have therapeutic consequences. The distinction between AD and psychiatric disorders is essential because (1) the prognosis and management of the patient are different, (2) psychiatric symptoms in AD can be the expression of unmet clinical needs requiring specific treatment while avoiding antipsychotic drugs and (3) some treatments are not recommended in neurodegenerative diseases but are widely used in psychiatric disorders, such as anticholinergic drugs [37–39]. It clearly appears really helpful to get a precise differentiation between AD and pure psychiatric disorders to give and adapt an appropriate treatment and to avoid deleterious therapeutics and management. Consequently, for the benefit of the patient and the patient’s family, the use of biomarkers improves the precision of the diagnosis and allows provision of better information and medical care to the patient as well as more specific supportive care for the patient’s relatives.

Some limitations of the present study have to be considered. First, there were two aspects of recruiting bias: (1) The participation of the memory clinics was on a voluntary basis, leading to overrepresentation of the clinics performing LP in routine clinical practice and in clinical research; and (2) patients were referred for cognitive disorders or atypical psychiatric disorders, probably explaining the high rate of patients with AD. A similar study in psychiatric centres might produce different results according to the age and clinical characteristics of the patients as well as differences in diagnostic procedures used in psychiatric consultations. In memory centres, clinicians have to be careful before concluding a diagnosis of psychiatric disorder and referring patients to a psychiatric centre.

A second limitation is that, because of refusal or medical contraindications, LP could not be performed in all patients from the referring centres. Third, even though previous studies indicated a good correlation between CSF results and neuropathology [13, 19–21], in this study we did not have pathological analysis available to confirm CSF findings. Further assessments using amyloid and tau PET imaging would certainly be worth trying. Fourth, psychiatric evaluations were not done using standardized interviews that would have allowed us to take into account atypical features and age at onset of the psychiatric disorders. Fifth, complementary data were recorded retrospectively in a subgroup of patients allowing to obtain follow up information but disadvantage to provide blinded (from CSF biomarker results) information.

## Conclusions

As the management and the prognosis of patients with psychiatric disorders and AD are really different, the results of the present study are very useful in underlining the importance of and need for complementary explorations when psychiatric disorders with cognitive decline are addressed in memory centres. Furthermore, the improvement of AD diagnosis will be important in the future when appropriate effective therapies become available and are routinely proposed to patients with AD.

## Abbreviations

AD, Alzheimer’s disease; BNA, Banque Nationale Alzheimer; CSF, cerebrospinal fluid; DLB, dementia with Lewy bodies; FTD, frontotemporal dementia; LP, lumbar puncture; MCI, mild cognitive impairment; PET, positron emission tomography
